# Supplementary material for: The care needs of persons with oropharyngeal dysphagia and their informal caregivers: A scoping review
Source: PLoS One. 2021 Sep 23;16(9):e0257683. doi: 10.1371/journal.pone.0257683 (PMC8460009; doi:10.1371/journal.pone.0257683)
Supplement: S1 Table — (DOCX) [file pone.0257683.s002.docx]

**S2 Table. Search strategies.**

| PubMed | |
| --- | --- |
| (‘Deglutition’[Mesh] OR ‘Deglutitions’ OR ‘Swallowing’ OR ‘Swallowings’ OR ‘Deglutition Disorders’[Mesh] OR ‘Deglutition Disorder’ OR ‘Disorders, Deglutition’ OR ‘Swallowing Disorders’ OR ‘Swallowing Disorder’ OR ‘Dysphagia’ OR ‘Oropharyngeal Dysphagia’ OR ‘Dysphagia, Oropharyngeal’) AND (‘Needs Assessment’[Mesh] OR ‘Needs Assessments’ OR ‘Unmet Need*’ OR ‘care need*’ OR ‘health need*’ OR ‘healthcare need*’ OR ‘support need*’ OR ‘caregiver need*’ OR ‘caregivers need*’ OR ‘caregiving need*’ OR ‘caring need*’ OR ‘perceived need*’ OR ‘patient need*’ OR ‘patients need*’ OR ‘personal need*’ OR ‘recipient need*’ OR ‘recipients need*’ OR ‘need satisfaction’ OR ‘needs satisfaction’ OR ‘spouse need*’ OR ‘family need*’) | |
| PsycInfo | |
| #1 | exp swallowing'/ |
| #2 | 'deglutition' OR 'oropharyngeal swallow' OR 'reflex' OR 'swallow' OR 'swallow function' OR 'swallow reflex' OR 'swallowing reflex' |
| #3 | exp Dysphagia/ |
| #4 | ('aphagopraxia' OR 'deglutition disorder' OR 'deglutition disorders' OR 'dysphagias' OR 'swallowing disorder').mp. [mp=title, abstract, heading word, table of contents, key concepts, original title, tests & measures, mesh] |
| #5 | 1 or 2 or 3 or 4 |
| #6 | exp Needs/ |
| #7 | exp Need Satisfaction/ |
| #8 | exp Needs Assessment/ |
| #9 | ('needs assessments' OR 'unmet need*' OR 'care need*' OR 'health need*' OR 'healthcare need*' OR 'support need*' OR 'caregiver need*' OR 'caregivers need*' OR 'caregiving need*' OR 'caring need*' OR 'perceived need*' OR 'patient need*' OR 'patients need*' OR 'personal need*' OR 'recipient need*' OR 'recipients need*' OR 'need satisfaction' OR 'needs satisfaction' OR 'spouse need*' OR 'family need*').mp. [mp=title, abstract, heading word, table of contents, key concepts, original title, tests & measures, mesh] |
| #10 | 6 or 7 or 8 or 9 |
| #11 | 5 and 10 |
| Embase* | |
| #1 | 'swallowing'/exp |
| #2. | 'deglutition' OR 'oropharyngeal swallow' OR 'reflex' OR 'swallow' OR 'swallow function' OR 'swallow reflex' OR 'swallowing reflex' |
| #3 | 'dysphagia'/exp OR 'dysphagia' |
| #4 | 'aphagopraxia' OR 'deglutition disorder' OR 'deglutition disorders' OR 'dysphagias' OR 'swallowing disorder' |
| #5 | #1 OR #2 OR #3 OR #4 |
| #6 | 'needs assessment'/exp OR 'needs assessment' |
| #7 | 'personal needs'/exp OR 'personal needs' |
| #8 | 'social needs'/exp OR 'social needs' |
| #9 | 'needs assessments' OR 'unmet need*' OR 'care need*' OR 'health need*' OR 'healthcare need*' OR 'support need*' OR 'caregiver need*' OR 'caregivers need*' OR 'caregiving need*' OR 'caring need*' OR 'perceived need*' OR 'patient need*' OR 'patients need*' OR 'personal need*' OR 'recipient need*' OR 'recipients need*' OR 'need satisfaction' OR 'needs satisfaction' OR 'spouse need*' OR 'family need*' |
| #10 | #6 OR #7 OR #8 OR #9 |
| #11 | #5 AND #10 |
| Wiley Cochrane Library | |
| #1 | MeSH descriptor: [Deglutition] explode all trees |
| #2 | Deglutitions OR Swallowing OR Swallowings |
| #3 | MeSH descriptor: [Deglutition Disorders] explode all trees |
| #4 | ‘Disorders, Deglutition’ OR ‘Swallowing Disorders’ OR ‘Swallowing Disorder’ OR ‘Deglutition Disorder’ OR Dysphagia OR ‘Dysphagia, Oropharyngeal’ OR ‘Oropharyngeal Dysphagia’ |
| #5 | #1 OR #2 OR #3 OR #4 |
| #6 | MeSH descriptor: [Needs Assessment] 2 tree(s) exploded |
| #7 | 'needs assessments' OR 'unmet need*' OR 'care need*' OR 'health need*' OR 'healthcare need*' OR 'support need*' OR 'caregiver need*' OR 'caregivers need*' OR 'caregiving need*' OR 'caring need*' OR 'perceived need*' OR 'patient need*' OR 'patients need*' OR 'personal need*' OR 'recipient need*' OR 'recipients need*' OR 'need satisfaction' OR 'needs satisfaction' OR 'spouse need*' OR 'family need*' |
| #8 | #6 OR #7 |
| #9 | #5 AND #8 |
| Cinahl | |
| S1 | ‘Deglutition’[Mesh] |
| S2 | (Deglutitions) OR (Swallowing) OR (Swallowings) |
| S3 | ‘Deglutition Disorders’[Mesh] |
| S4 | ‘Disorders, Deglutition’ OR ‘Swallowing Disorders’ OR ‘Swallowing Disorder’ OR ‘Deglutition Disorder’ OR Dysphagia OR ‘Dysphagia, Oropharyngeal’ OR ‘Oropharyngeal Dysphagia’ |
| S5 | S1 OR S2 OR S3 OR S4 |
| S6 | ‘Needs Assessment’ [Mesh] |
| S7 | 'needs assessments' OR 'unmet need*' OR 'care need*' OR 'health need*' OR 'healthcare need*' OR 'support need*' OR 'caregiver need*' OR 'caregivers need*' OR 'caregiving need*' OR 'caring need*' OR 'perceived need*' OR 'patient need*' OR 'patients need*' OR 'personal need*' OR 'recipient need*' OR 'recipients need*' OR 'need satisfaction' OR 'needs satisfaction' OR 'spouse need*' OR 'family need*' |
| S8 | S6 OR S7 |
| S9 | S5 AND S8 |

* The search was conducted on Embase records only
